# Polyploidization Redirects Carbon Flux to Diterpenoid Biosynthesis in *Nicotiana sylvestris*

**DOI:** 10.3390/plants15142158

**Published:** 2026-07-13

**Authors:** Xiuming Wu, Kexin Chen, Changqing Yang, Yangyang Sun, Min Ren

**Affiliations:** Tobacco Research Institute, Chinese Academy of Agricultural Sciences, Qingdao 266101, China

**Keywords:** *Nicotiana sylvestris*, polyploidization, metabolic reprogramming, carbon flux, diterpenoid biosynthesis

## Abstract

Polyploidization frequently rewires plant metabolism, leading to increased biomass and altered metabolic profiles. This makes it a promising strategy for crop improvement and breeding. However, the mechanism underlying this metabolic reprogramming remains largely unknown. In this study, we generated three stable autotetraploid *Nicotiana sylvestris* lines through colchicine treatment of diploids followed by continuous selection. Comparative metabolomic and transcriptomic analysis revealed 467 and 167 differential accumulated metabolites, as well as 587 and 154 differentially expressed genes between tetraploids and diploids in leaves and flowers, respectively. The increased expression levels of Calvin–Benson cycle genes and the elevated contents of pyruvate and glyceraldehyde-3-phosphate indicated enhanced photosynthetic carbon assimilation in tetraploid plants. Notably, the amounts of diterpenoids, such as cembratrienediol (CBT-diol) and cis-abienol, increased dramatically in tetraploids, whereas sucrose, the major product of photosynthesis, was markedly depleted. Consistent with this, the expression levels of the methylerythritol phosphate pathway and diterpene biosynthesis genes were upregulated, while *sucrose phosphate synthase* was downregulated in autotetraploid plants. These results suggest that polyploidization enhanced carbon assimilation and redirects the increased metabolic flux towards diterpenoid biosynthesis, rather than carbohydrate metabolism. Our findings provide new insight into the metabolic switch orchestrated by polyploidization, establish a mechanistic framework for polyploidy-driven metabolic innovation, and highlight autopolyploidization as a promising strategy for metabolic engineering and crop improvement.

## 1. Introduction

Polyploidy, the possession of more than two complete sets of chromosomes in a nucleus, is a widespread and influential evolutionary phenomenon in the plant kingdom [1]. It has long been recognized as a major driver of diversification, speciation and adaptation, contributing to genomic novelty and phenotypic plasticity [2,3]. Based on the origin of duplicated chromosome sets, polyploids are classified into autopolyploids, which originate from intraspecific whole-genome duplication (WGD) within a single species, and allopolyploids, which are derived from interspecific hybridization followed by genome doubling [2]. A survey of 47 vascular plant genera revealed that 76% of plant species are diploids and 24% are polyploids, comprising 11% allopolyploids and 13% autopolyploids [4]. Moreover, polyploidy has facilitated the domestication of agricultural plants: approximately 54% of economically important crop species are polyploid, including wheat (*Triticum aestivum*), cotton (*Gossypium hirsutum*), sugarcane (*Saccharum officinarum*), potato (*Solanum tuberosum*), oilseed rape (*Brassica napus*) and tobacco (*Nicotiana tabacum*) [5].

While both forms of polyploidy contribute to plant evolution and crop domestication, autopolyploids play indispensable roles in plant diversification and speciation, as they arise solely from genome duplication within a single species, thereby altering genome structure and function by increasing gene dosage, promoting genome restructuring, and creating opportunities for functional divergence among duplicated genes [6]. A common consequence of genome doubling is an increase in cell size, which often manifests as enlarged organs and enhanced vegetative growth [7]. Many crops, including banana (triploid derived from *Musa acuminata*) and alfalfa (tetraploid of *Medicago sativa*), owe their superior agronomic traits to autopolyploidy [8,9,10,11]. Nevertheless, despite its evolutionary and agricultural importance, how WGD reshapes plant growth, development, and physiological traits remains elusive.

At the molecular level, one of the most conspicuous consequences of homologous chromosome doubling is the global increase in gene dosage, which can trigger diverse transcriptional responses, including nonadditive expression (deviations from the parental mean), expression level dominance, transgressive expression, and allele-specific expression bias [12,13]. Comparative transcriptomic studies across diverse plant lineages have revealed that such changes in gene expression are common features of neopolyploids, often driving altered transcriptional output, cellular metabolism and phenotypic variations such as increased cell and organelle size, and enhanced vegetative growth [14,15,16]. For example, genome doubling of watermelon (*Citrullus lanatus*) lead to enlarged organs, increased content of secondary metabolites, and enhanced tolerance to biotic and abiotic stresses [17,18,19], and these phenotypic shifts are associated with a large number of upregulated genes and differentially methylated regions in their promoters [19]. In contrast, in autotetraploid rice (*Oryza sativa*), increased DNA methylation of class II transposable elements (TEs) is thought to suppress the expression of neighboring genes, resulting in minimal transcriptome alterations from its diploid progenitor [20]. Integrative RNA-seq and Hi-C analysis of diploid (AA) and artificial autotetraploid (AAAA) *Brassica rapa* revealed thousands of differentially expressed genes (DEGs), including 15 in activated and 80 in repressed topologically associating domains (TADs), linking transcriptional changes to high-order genomic structural alterations [21]. These studies demonstrate that the transcriptional consequences of WGD are highly context-dependent and involve multiple layers of genomic regulation; however, little is known about the cellular and molecular mechanisms underpinning the superiority of autopolyploid plants. Moreover, despite extensive documentation of morphological and physiological changes associated with polyploidy, several critical questions remain elusive, e.g., how homologous genome duplication reprograms gene expression and metabolic networks in plants, whether the relatively modest transcriptional perturbations observed in autopolyploids are sufficient to drive the substantial metabolic rewiring that underpins phenotypic novelty, and the identities of the central metabolic hubs that translate gene dosage effects into systematic phenotypic variation. Another poorly understood dimension is the tissue-specific responses to polyploidization, particularly the coordination between source organs (e.g., leaves) and sink organs (e.g., roots or flowers), which is essential for whole plant physiological integration.

The Solanaceae family includes approximately 90 genera and 3000~4000 species, many of which are essential crops, medicinal and ornamental plants, such as potato (*S. tuberosum*), tomato (*Solanum lycopersicum*), eggplant (*Solanum melongena*), deadly nightshade (*Atropa belladonna*), mandrake (*Mandragora officinarum*), petunia (*Petunia × hybrida*) and velvet tongue (*Salpiglossis sinuata*) [22]. The *Nicotiana* genus comprises 76 naturally occurring species across 13 sections, predominantly native to North and South America and Australia, including the well-known tobacco (*Nicotiana tabacum*), an allotetraploid crop cultivated in over 120 countries [23]. The diploid species *Nicotiana sylvestris* (2n = 2x = 24) is not only an ornamental plant due to its distinctive floral and vegetative morphology, but also the maternal progenitor of tobacco (2n = 4x = 48), having hybridized with *Nicotiana tomentosiformis* (2n = 2x = 24) in the Andes within the last 200,000 years [24]. With a high-quality reference genome and established nuclear and plastid transformation protocols, *N. sylvestris* has emerged as a robust model for studying plant metabolism and metabolic engineering [24,25,26,27,28,29]. Its relatively compact diploid genome, evolutionary relationship to the polyploid crop tobacco, and ability to accumulate high levels of diterpenoids make it an ideal platform for dissecting the consequences of polyploidization.

In this study, we generated stable autotetraploid *N. sylvestris* lines and performed comparative multi-omics analysis of diploid and tetraploid plants. Our objective was to elucidate how polyploidization reshapes gene expression and metabolic networks. We reveal that polyploidization induces extensive metabolic reprogramming and identify a central metabolic switch in which carbon flux is redirected from sucrose biosynthesis towards diterpenoid production. These findings provide new insights into the molecular basis of polyploid-induced phenotypic variation and highlight potential targets for exploiting polyploidy in crop improvement and metabolic engineering.

## 2. Results

### 2.1. Generation and Characterization of Autotetraploid N. sylvestris Lines

**Figure 1:**
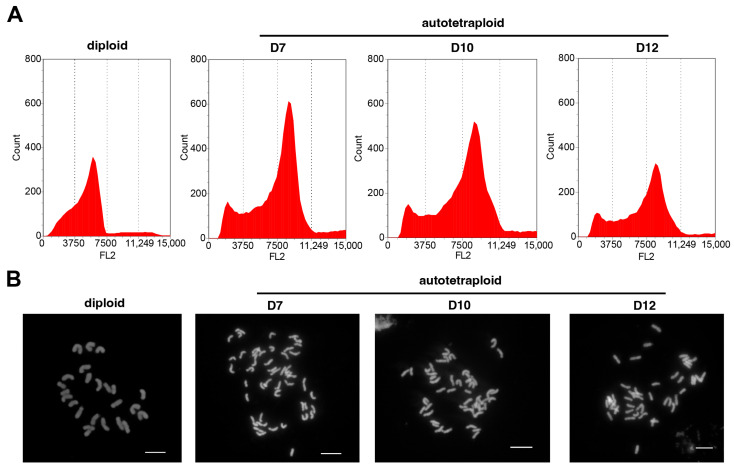
Ploidy analysis of diploid and autotetraploid *N. sylvestris* plants. (**A**) Flow cytometric analysis of diploid and autotetraploid plants (primary lines, or 1st generation). (**B**) Chromosomes in root cells of diploid and autotetraploid plants (3rd generation). Bar = 5 μm.

We employed a tissue culture-based method to generate autoploid plants. Diploid *N. sylvestris* seeds were surface-sterilized and germinated on Murashige & Skoog (MS) medium. Leaf disks excised from three-month-old plants were treated with 1 mM colchicine for 3 h and then cultivated for shoot and root regeneration. Totally 23 regenerated plantlets were obtained and subsequently transferred to soil for normal growth. To identify tetraploid individuals, nuclei were extracted from young leaves of each regenerated plant and analyzed through flow cytometry. The shift in fluorescence intensity peak position from ~5000 in diploids to ~10,000 indicated substantial increase in DNA content in tetraploid plants (Figure 1A). Among the regenerated plants, 16 tetraploid individuals were identified as primary lines (designated as D1–D16). In contrast, although 15 plants were regenerated from the control treatment (sterilized water), no tetraploid plant was identified.

To ensure genomic stability and exclude chimeric individuals, seeds collected from lines D1–D16 were sown in soil, and their progeny were screened through flow cytometric analysis. Only lines producing 100% tetraploid progeny were retained, and seeds from these individuals were propagated to the next generation. After three successive generations of selection, three stable autotetraploid lines (D7, D10 and D12) were obtained. Chromosome counting analysis in root cells confirmed that these tetraploid plants possessed 48 chromosomes, in contrast to the 24 chromosomes in diploid plants (Figure 1B).

**Figure 2:**
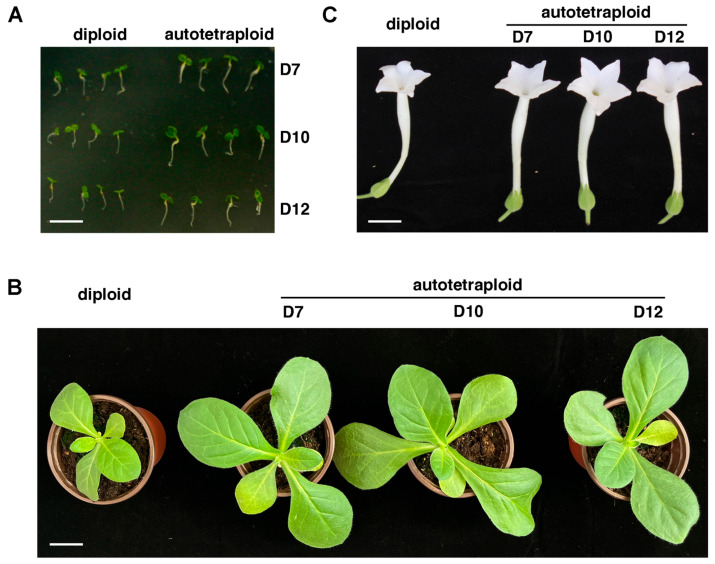

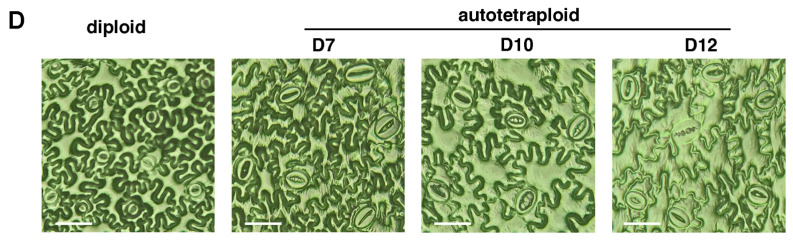
Morphological comparison of diploid and tetraploid *N. sylvestris* plants. (**A**) Seedlings at 10 days post germination. Bar = 1 cm. (**B**) One-month-old plants. Bar = 5 cm. (**C**) Flowers from 5-month-old plants. Bar = 1 cm. (**D**) Epidermal cells and stomata on the abaxial surface of leaves. Bar = 50 μm.

To evaluate the effects of genome doubling on plant growth, diploid and tetraploid plants were germinated on MS medium and then transplanted to soil. Pronounced morphological differences were observed between diploid and tetraploid plants (Figure 2). Compared with diploids, tetraploid plants exhibited significantly larger seedlings (two-week-old, Figure 2A) and young plants (one-month-old, Figure 2B), indicating accelerated early growth through polyploidization. Flowers of five-month-old tetraploid plants were also substantially larger than those of diploids (Figure 2C). Additionally, stomata were markedly larger in tetraploid leaves, a typical manifestation of the ‘gigas effect’ associated with increased DNA content (Figure 2D).

### 2.2. Metabolome Analysis of Diploid and Tetraploid N. sylvestris

**Figure 3:**
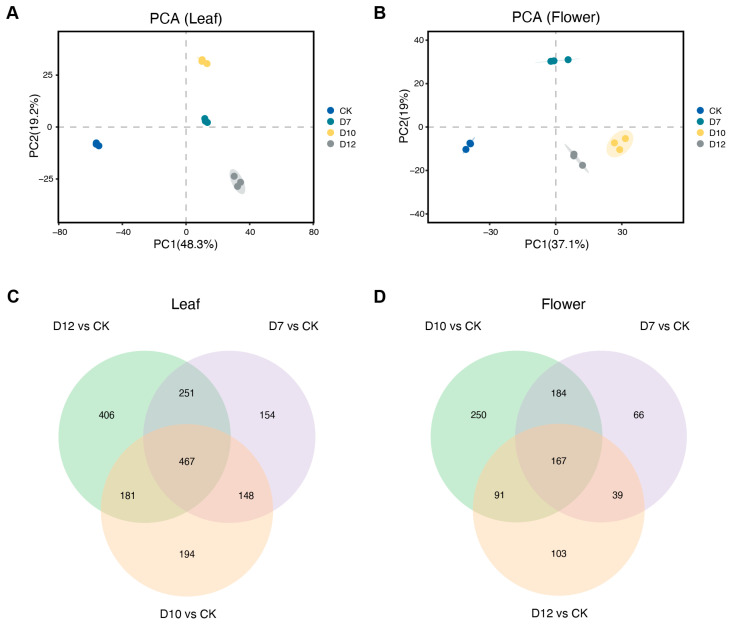
Metabolomic analysis of diploid and tetraploid *N. sylvestris*. (**A**,**B**) PCA of metabolomic profiles of leaves (**A**) and flowers (**B**) of diploid and tetraploid plants (D7, D10 and D12). Shaded ellipses represent 95% confidence intervals. (**C**,**D**) Venn diagrams illustrating specific and shared DAMs among D7 vs. CK, D10 vs. CK and D12 vs. CK comparisons in leaves (**C**) and flowers (**D**).

To further assess the impacts of genome duplication on metabolism, leaves and flowers were collected from five-month-old diploid and tetraploid plants and subjected to untargeted metabolomic profiling. A total of 3923 and 3528 metabolites were annotated in leaves and flowers separately through comparison with metabolite databases. Both principal component analysis (PCA) and partial least squares discriminant analysis (PLS-DA) revealed a clear separation between diploid and autotetraploid individuals in both leaves and flowers (Figure 3A,B and Figure S1), indicating that polyploidization results in significant metabolic profile changes in both organs.

Comparative analysis of differentially accumulated metabolites (DAMs, |log_2_(FoldChange)| > 1, VIP > 1, *p* < 0.05) between autotetraploid and diploid plants revealed substantial differences. In leaves, 1020 (791 down, 229 up), 990 (699 down, 291 up) and 1305 (1086 down, 219 up) DAMs were identified in lines D7, D10 and D12, respectively (Figure S2A–C). In flowers, 456 (109 down, 347 up), 692 (285 down, 407 up) and 400 (179 down, 221 up) DAMs were detected in D7, D10 and D12, respectively (Figure S2D–F). These results suggest not only that substantially more DAMs were detected in leaves than in flowers, but also reveal a striking tissue-specific pattern: in leaves, the majority of DAMs were downregulated, whereas in flowers, most DAMs were upregulated. Among these, 467 and 167 metabolites were shared across all three lines comparisons in leaves and flowers, respectively (Figure 3C,D), representing robust and ploidy-dependent metabolic signatures.

**Figure 4:**
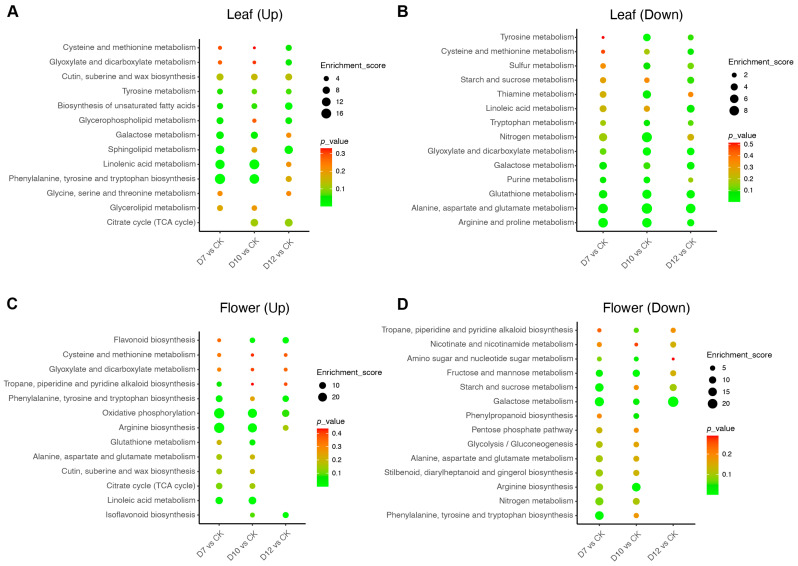
KEGG enrichment analysis of DAMs between diploid and tetraploid *N. sylvestris* plants. (**A**) Enrichment analysis of upregulated DAMs in leaves of tetraploids. (**B**) Enrichment analysis of downregulated DAMs in leaves of tetraploids. (**C**) Enrichment analysis of upregulated DAMs in flowers of tetraploids. (**D**) Enrichment analysis of downregulated DAMs in flowers of tetraploids.

The Kyoto Encyclopedia of Genes and Genomes (KEGG) annotation and pathway enrichment analysis of these shared DAMs revealed distinct metabolic reprogramming patterns. In leaves, amino acid metabolism was changed (increase and decrease) significantly (Figure 4A,B), while in flowers, specialized metabolism, including alkaloids and flavonoids, were changed dramatically (Figure 4C,D). Importantly, metabolites associated with carbohydrate and energy metabolism, especially starch and sucrose metabolism, were decreased in both leaves and flowers of tetraploid plants (Figure 4).

### 2.3. Transcriptomic Comparison of Diploid and Tetraploid N. sylvestris

**Figure 5:**
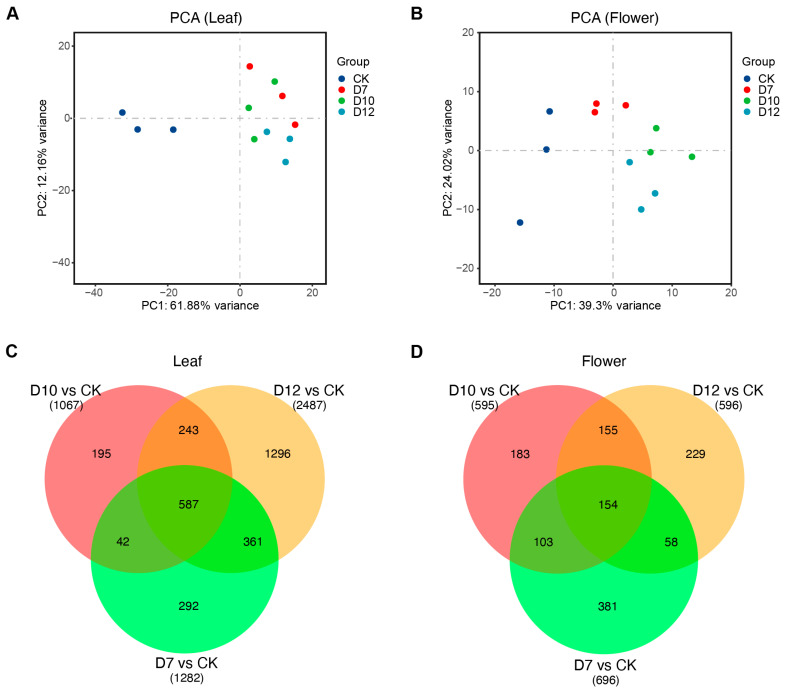
Transcriptomic analysis of diploid and tetraploid *N. sylvestris*. (**A**,**B**) PCA of transcriptomic profiles of leaves (**A**) and flowers (**B**) of diploid and tetraploids (D7, D10 and D12). (**C**,**D**) Venn diagrams illustrating specific and shared DEGs among D7 vs. CK, D10 vs. CK and D12 vs. CK comparisons in leaves (**C**) and flowers (**D**).

To further investigate the impact of polyploidization on gene expression, we performed RNA-seq analysis of leaves and flowers from diploid and tetraploid plants. The PCA of global transcript abundance revealed a clear separation between diploid and tetraploid replicate samples, with the first two principal components accounting for about 74% and 64% of the total variances in their leaves and flowers, respectively (Figure 5A,B). In leaves, 1282 (903 down, 379 up), 1067 (765 down, 302 up) and 2487 (1369 down, 1118 up) DEGs (|Log_2_(FoldChange)| > 1, q-value < 0.05) were identified in D7, D10 and D12 (Figure S3A–C), with 587 DEGs common to all three comparisons (Figure 5C). In flowers, 696 (274 down, 422 up), 595 (182 down, 413 up) and 596 (210 down, 386 up) DEGs were detected, respectively (Figure S3D–F), among which 154 genes were shared across all comparisons (Figure 5D). Consistent with the DAM profiles, substantially more DEGs were detected in leaves than in flowers, and a tissue-specific directional bias was evident: the majority of DEGs were downregulated in leaves, whereas upregulated in flowers.

**Figure 6:**
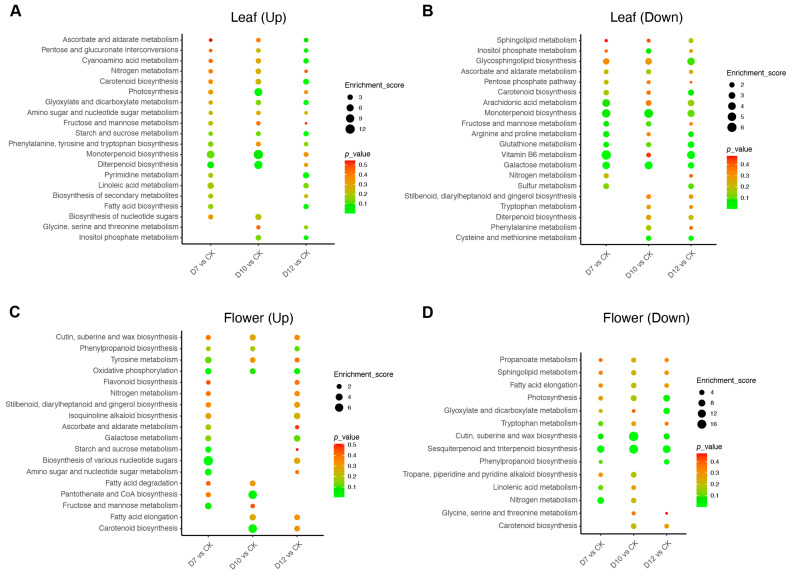
KEGG enrichment analysis of DEGs between diploid and tetraploid *N. sylvestris* plants. (**A**) Enrichment analysis of upregulated DEGs in leaves of tetraploids. (**B**) Enrichment analysis of downregulated DEGs in leaves of tetraploids. (**C**) Enrichment analysis of upregulated DEGs in flowers of tetraploids. (**D**) Enrichment analysis of downregulated DEGs in flowers of tetraploids.

The KEGG enrichment analysis of the common DEGs revealed that in leaves, genes involved in diterpenoid metabolism, nitrogen metabolism and the pentose phosphate pathway were upregulated, whereas those associated with amino acid metabolism increased (Figure 6A,B). In flowers, genes involved in phenylpropanoid biosynthesis were upregulated, whereas those involved in sesquiterpenoid metabolism were downregulated (Figure 6C,D).

### 2.4. Elevated Diterpenoid Metabolism in Tetraploid N. sylvestris Leaves

**Figure 7:**
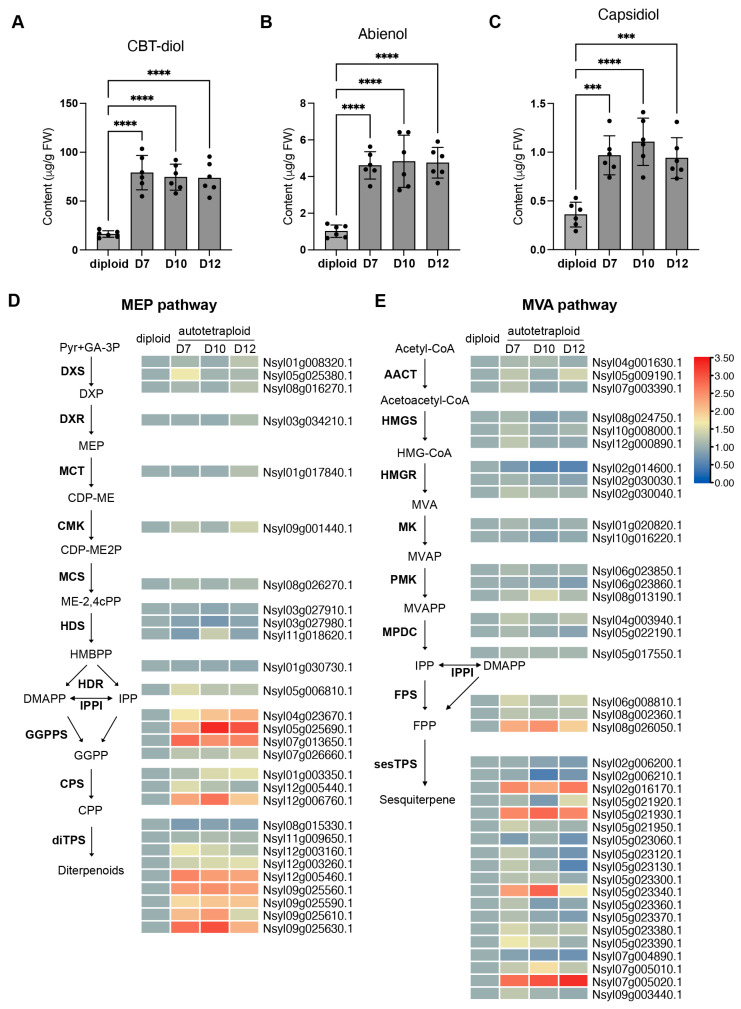
Enhanced terpenoid biosynthesis in leaves of tetraploid *N. sylvestris*. (**A**–**C**) Contents of CBT-diol (**A**), cis-abienol (**B**) and capsidiol (**C**) in diploid and tetraploid *N. sylvestris* leaves. ***, *p* < 0.001; ****, *p* < 0.0001. (**D**) Heatmap of expression of MEP pathway and diterpenoid biosynthesis genes (in Log_2_FC). (**E**) Heatmap of expression of MVA pathway and sesquiterpenoid biosynthesis genes (in Log_2_FC).

*N. sylvestris* is known to accumulate substantial quantities of diterpenoids. Since both metabolomic and transcriptomic analysis indicated significant increase in diterpenoid metabolism in tetraploid plants, we performed quantitative comparison of terpenoid profiles between diploid and tetraploid plants. Gas chromatography–mass spectrum (GC-MS) analysis of leaf hexane extracts revealed that the contents of diverse terpenoids, including diterpenoids of cembratriene-diol (CBT-diol) and cis-abienol, along with the sesquiterpenoid phytoalexin capsidiol, increased dramatically in tetraploid plants (Figure 7A–C).

This massive accumulation was supported by coordinated transcriptional upregulation of the biosynthetic machinery. In plants, diterpenoids are synthesized via the plastidial methylerythritol phosphate (MEP) pathway, which converts pyruvate and glyceraldehyde-3-phosphate (GAP) to isopentenyl pyrophosphate (IPP) and dimethylallyl pyrophosphate (DMAPP). They are subsequently condensed by geranylgeranyl diphosphate synthase (GGPPS) to form geranylgeranyl diphosphate (GGPP) for diterpene biosynthesis [30]. As shown in Figure 7D, expression of MEP pathway genes, including the rate-limiting *1-deoxy-D-xylulose-5-phosphate synthase* (*DXS*), was increased dramatically in tetraploids. Additionally, transcript levels of *GGPPS* and most diterpene synthases were also upregulated significantly, consistent with the observed accumulation of diterpenoids.

The cytosolic mevalonate (MVA) pathway begins with the condensation of acetyl-CoA and produced IPP and DMAPP as substrates for the production of sesquiterpenes. As shown in Figure 7E, the expression of many MVA pathway genes were upregulated in tetraploid plants, such as *acetyl-CoA acetyltransferase* (*AACT*), *3-hydroxy-3-methylglutaryl-Coenzyme A synthase* (*HMGS*) and *3-hydroxy-3-methylglutaryl-coenzyme A reductase* (*HMGR*). While most sesquiterpene synthase genes were unchanged, with the notable exception of genes encoding 5-epi-aristolochene synthase (EAS), the key enzyme for capsidiol biosynthesis, was specifically induced, consistent with the elevated capsidiol levels.

### 2.5. Elevated Carbon Assimilation and Decreased Sucrose Biosynthesis in Tetraploids

**Figure 8:**
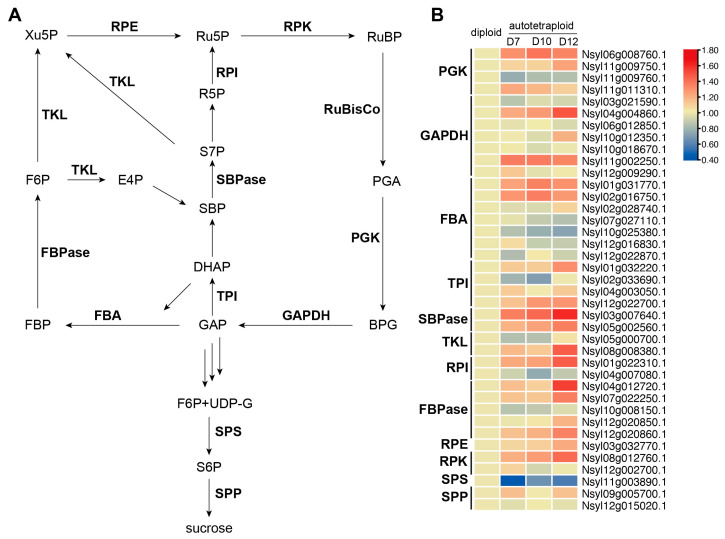

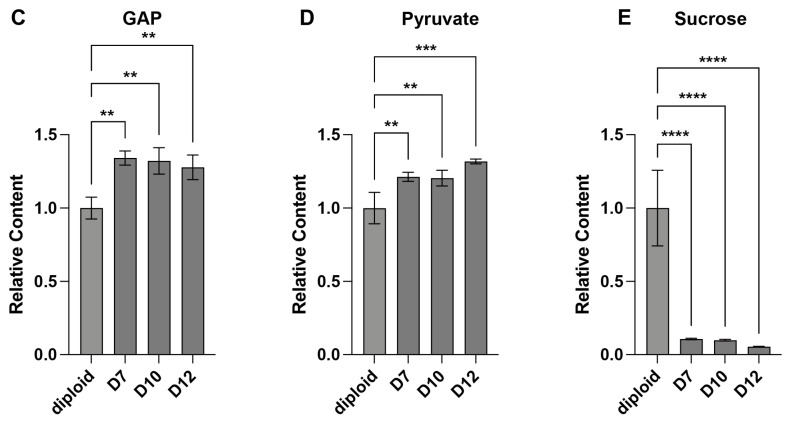
Elevated carbon assimilation and decreased sucrose biosynthesis in leaves of diploid and tetraploid *N. sylvestris*. (**A**) Schematic diagram of Calvin–Benson cycle and sucrose biosynthetic pathway. Arrows represent biochemical reactions catalyzed by corresponding enzymes. Ru5P: ribulose 5-phosphate; RuBP: ribulose 1,5-bisphosphate; PGA: 3-phosphoglycerate; BPG: 1,3-bisphosphoglycerate; GAP: glyceraldehyde-3-phosphate; DHAP: dihydroxyacetone phosphate; FBP: fructose 1,6-bisphosphate; F6P: fructose 6-phosphate; E4P: erythrose 4-phosphate; Xu5P: xylulose 5-phosphate; SBP: sedoheptulose 1,7-bisphosphate; SBP: sedoheptulose 1,7-bisphosphate; S7P: sedoheptulose 7-phosphate; R5P: ribose 5-phosphate; Ru5P: ribulose 5-phosphate; UDP-G: UDP-glucose; S6P: sucrose 6-phosphate; RuBisCO: ribulose-1,5-bisphosphate carboxylase/oxygenase; PGK: phosphoglycerate kinase; GAPDH: glyceraldehyde-3-phosphate dehydrogenase; FBA: fructose-bisphosphate aldolase; TPI: triose phosphate isomerase; SBPase: sedoheptulose-1,7-bisphosphatase; TKL: transketolase; RPI: ribose 5-phosphate isomerase; FBPase: fructose bisphosphatase; RPE: ribulose 5-phosphate epimerase; RPK: phosphoribulokinase; SPS: sucrose phosphate synthase; SPP: sucrose phosphate phosphatase. (**B**) Heatmap of expression of Calvin–Benson cycle and sucrose biosynthetic genes (in Log_2_FC). (**C**–**E**) Relative contents of GAP (**C**), pyruvate (**D**) and sucrose (**E**) in leaves of diploid and tetraploids. **, *p* < 0.01; ***, *p* < 0.001; ****, *p* < 0.0001.

The MEP pathway utilizes pyruvate and GAP as precursors for the synthesis of diterpenoids. The dramatic increase in diterpenoid accumulation indicates increased demand for these metabolites, which are derived from photosynthetic carbon assimilation in plastids. We analyzed the expression of genes in the Calvin–Benson cycle (Figure 8A), most of which were significantly upregulated in tetraploid plants (Figure 8B), suggesting that polyploidization improved carbon assimilation capacity. Consistent with this, the contents of pyruvate and GAP increased significantly in tetraploid leaves (Figure 8C,D). However, the content of sucrose, the principal end-product of photosynthetic carbon fixation and the major transported carbohydrate in plants, decreased by more than 80% in tetraploid leaves (Figure 8E). Consistently, expression of the key biosynthetic gene *Sucrose Phosphate Synthase* (*SPS*) decreased significantly in tetraploid plants, indicating that reduced sucrose content was associated with decreased biosynthesis rather than enhanced catabolism or transport.

## 3. Discussion

Polyploidization is a pervasive evolutionary force that drives genomic innovation and phenotypic diversity in plants. However, the mechanisms by which polyploidization rewires metabolic networks remain poorly understood. In this study, we generated stable autotetraploid lines of *N. sylvestris* and performed integrated multi-omics profiling analysis. The coordinated changes in gene expression and metabolite accumulation, validated across three independent tetraploid lines, provide a mechanistic framework for understanding polyploidy-driven metabolic innovation.

Previous comparative studies of autopolyploid plants have revealed highly variable transcriptional consequences, ranging from minimal changes in autotetraploid rice [20] to thousands of DEGs in watermelon and *Brassica rapa* [19,21]. In this study, we identified a robust set of 587 DEGs in leaves and 154 DEGs in flowers common to all three tetraploid lines, indicating that meaningful and reproducible transcriptional reprogramming occurs upon genome doubling. The relatively large number of DEGs in leaves compared with flowers may reflect a greater sensitivity of source tissue metabolism to ploidy-induced changes in gene dosage and cellular architecture. It is also possible that epigenetic alterations, such as changes in DNA methylation or chromatin conformation reported in other autopolyploid systems [19,20], contribute to the tissue-specific expression divergence. Future studies examining the methylome and chromatin accessibility in these tetraploid lines will help elucidate the upstream regulatory layers driving the transcriptional shifts.

Compared to diploid plants, polyploid plants generally exhibit more vigorous growth, larger size, and improved environmental adaptability [31,32,33,34,35,36,37,38,39]. In addition to morphological changes, polyploidy also alters contents of specialized metabolites and thus is used to generate medicinal plants or crops with larger organs and improved ingredients. For example, polyploidization of *Catharanthus roseus* not only caused significant changes in the changes in plant height, leaf length and width, plant fresh and dry weight, stem and flower diameter, but also increased the contents of anticancer compounds vincristine, vinblastine, catharanthine and vindoline [40]; in *Lycium chinense*, polyploidization increased the contents of carotenoids such as lutein, β-carotene, neoxanthin and violaxanthin [41]; and in *Stevia rebaudiana*, the content of steviol glycosides (including rebaudioside A, rebaudioside C and steviolbioside), along with their precursor steviol, increased significantly after polyploidization [42]. In our study, the most prominent metabolic signatures in autotetraploid *N. sylvestris* was the dramatic increase in diterpenoid accumulation, such as CBT-diol and cis-abienol. Transcriptomic analysis showed that the MEP pathway genes, such as *DXS* and *GGPPS*, were significantly upregulated in leaves of tetraploid plants (Figure 7). Similarly, genes of the MVA pathway, such as *AACT*, *HMGS* and *HMGR*, were also upregulated. Notably, although most sesquiterpene synthase genes showed no significant expression change, *EAS* showed dramatic upregulation, leading to elevated accumulation of phytoalexin capsidiol (Figure 7). This concerted activation of both the MEP and MVA pathways, together with the selective activation of specific enzymes, suggests that polyploidization activates distinct specific metabolic modules. Given that CBT-diol plays vital roles in defensing against aphid and pathogens like *Spodoptera frugiperda* and *Phytophthora nicotianae* [43,44], cis-abienol inhibits *Ralstonia solanacearum* growth and enhances plant resistance to bacterial wilt [45], and capsidiol is a compound related to fungi resistance [46]. This metabolic shift towards phytoalexins may confer enhanced defensive capability, which may assist in improving their ecological adaptation. Moreover, *N. sylvestris* is the diploid progenitor of the allotetraploid crop tobacco (*N. tabacum*), and both species are known for their capacity to produce abundant diterpenoids. Our findings that autopolyploidization can dramatically upregulate terpenoid metabolism suggest that the duplicated genome may have provided a genomic environment permissive for enhanced specialized metabolism, which could have been further fixed and refined during domestication of tobacco, and contribute to the pathogen resistance and flavor profile of this economic crop [47].

While polyploidization is known to alter metabolic profiles, whether it can redirect metabolic flux between primary and secondary metabolism remains largely unknown. Here, we observed that in contrast to the dramatic increase in terpenoid biosynthesis, the content of sucrose, the principal end-product of photosynthetic carbon fixation and the major transported carbohydrate in plants was decreased in autotetraploid *N. sylvestris* plants. Both diterpenoids and sucrose are derived from the Calvin–Benson cycle: the MEP pathway uses pyruvate and glyceraldehyde-3-phosphate to produce isoprenoid precursors, whereas the sucrose biosynthesis utilizes triose-phosphate translocated from plastid to cytosol [30,48]. The decreased expression levels of genes encoding sucrose phosphate synthase, as well as the elevated contents of pyruvate and glyceraldehyde-3-phosphate, indicates a coordinated metabolic reprogramming of carbon allocation from primary to specialized metabolism, thereby enhancing accumulation of bioactive specialized metabolites. To the best of our knowledge, this is the first report of metabolic flux redirection induced by polyploidization.

In summary, our integrated multi-omics analysis demonstrates that polyploidization reprograms primary and specialized metabolism in *N. sylvestris* by shifting carbon flux from sucrose to terpenoid biosynthesis, accompanied by tissue-specific changes in gene expression and metabolite levels. The involvement of coordinated transcriptional regulators and metabolite transporters points to a sophisticated regulatory network that translates genome duplication into metabolic novelty. These results expand our understanding of the molecular basis of polyploidy-induced phenotypic variation and highlight the potential of exploiting polyploidy as a tool for metabolic engineering and crop improvement. Future work dissecting the cis-regulatory and epigenetic mechanisms that control the polyploidy-responsive regulon will be essential to fully harness this potential.

## 4. Materials and Methods

### 4.1. Plant Materials and Growth Conditions

Seeds of diploid and autotetraploid *N. sylvestris* were surface-sterilized with 70% (*v*/*v*) ethanol for 1 min, followed by 10% (*v*/*v*) H_2_O_2_ for 10 min, and then rinsed four times with sterile water. Sterilized seeds were germinated on an MS medium in a growth chamber at 25 °C under a 16/8 h (light/dark) photoperiod. Two-week-old seedlings were transplanted into growth substrate (Pindstrup Blond Gold, Syddjurs, Midtjylland, Denmark) and cultivated in a greenhouse under a photoperiod of 16/8 h (light/dark) at 25 °C. Leaf and flower samples were collected from five-month-old plants for transcriptomic and metabolomic analysis. Each experimental group comprised three independent diploid or tetraploid plants (three biological replicates).

### 4.2. Generation of Autotetraploid Plants Using Colchicine Treatment

Fifty seeds of diploid *N. sylvestris* were surface-sterilized and germinated on an MS medium as described above, and 37 plants were obtained. Leaf disks (1 cm × 1 cm) excised from a three-month-old plant (three leaf disks from each plant) were soaked in 1 mM colchicine solution for 3 h (treatment of sterilized water as control), then washed four times with sterilized water. The leaf disks were transferred to the shooting medium (MS + 30 g/L sucrose + 1.0 mg/L 6-BA + 8.0 g/L agar, pH 6.2) and cultivated for two months. Regenerated shoots were cut from the plantlets, transferred to rooting medium (MS + 30 g/L sucrose + 0.1 mg/L NAA + 8.0 g/L agar, pH 6.2), and cultivated for 1 month. Ultimately 23 regenerated plants were transplanted into growth substrate and grown in a greenhouse. The ploidy of each plant was determined through flow cytometric analysis, and those tetraploid plants were retained as independent lines (16 lines, D1–D16). Seeds collected from each line were sown in soil, and at least 10 plants from each line were analyzed for ploidy. This selection process was repeated for three consecutive generations to obtain stable lines D7, D10 and D12.

### 4.3. Flow Cytometric Analysis

Young leaves collected from diploid and tetraploid plants were immersed in 0.8 mL of pre-cooled nuclei isolation buffer (45 mM MgCl_2_, 20 mM 3-(N-morpholino) propanesulfonic acid (MOPS), 30 mM sodium citrate, 1% polyvinylpyrrolidone (PVP-40), 0.2% Triton X-100, 10 mM Na_2_EDTA, 20 μL/mL β-mercaptoethanol, pH 7.5), rapidly chopped with a razor blade and incubated on ice for 10 min. The nuclei suspension was obtained by filtering the homogenate through a 40 μm nylon mesh. Following the addition of propidium iodide (50 μg/mL), the suspension was incubated on ice for 1 h. Stained nuclei were analyzed using a BD FACScalibur flow cytometer (BD Biosciences, Franklin Lakes, NJ, USA) with 488 nm blue light excitation. For each sample, 10,000 events were recorded, with the coefficient of variation (CV) maintained < 5%. The data was analyzed using Modifit 3.0 software.

### 4.4. Chromosome Observation

The ploidy of the diploid and tetraploid plants was confirmed through chromosome observation. Roots excised from 1-month-old plants were pretreated in a saturated solution of 1,4-dichlorobenzene for 1 h, rinsed with distilled water, and fixed in Carnoy’s solution (glacial acetic acid: ethanol = 1:3, *v*/*v*) at 4 °C for 24 h. The fixed roots were dissociated in 38% HCl for 15 min, washed three times with distilled water, then squashed on a glass slide in a drop of carbol-fuchsin solution and covered with a coverslip. Chromosomes were observed and photographed under a 100× oil lens using an Olympus BX53 microscope (Olympus, Tokyo, Japan). Chromosome numbers were counted in 10 randomly selected cells per sample.

### 4.5. Analysis of Abaxial Epidermal Cell and Stomatal Characteristics

Fully expanded leaves collected from diploid and tetraploid plants were used for epidermal and stomatal observation. Leaf segments containing the main veins were fixed in the FAA solution (38% formaldehyde:glacial acetic acid:70% ethanol = 1:1:18, *v*/*v*/*v*) for 24 h, rinsed three times with water and blotted dry on filter paper. Epidermal cells and stomatal observations were performed using an Olympus BX53 microscope equipped with a 20× objective.

### 4.6. Transcriptome Analysis

Leaf and flower tissues were collected from five-month-old plants, frozen immediately in liquid nitrogen and ground into a fine powder. Total RNA was extracted using TRIzol reagent (Invitrogen, 15596018, Carlsbad, CA, USA) according to the manufacturer’s protocol. Briefly, TRIzol reagent (1 mL) was added to 100 mg of sample powder, vortexed vigorously, and incubated at room temperature for 5 min. Chloroform (200 μL) was added, mixed vigorously, incubated at room temperature for 2 min, and centrifuged at 12,000× *g* for 15 min at 4 °C. The aqueous phase (about 500 μL) was transferred to a 1.5 mL tube, mixed with equal volume of isopropanol, incubated at −20 °C for 1 h, and centrifuged at 12,000× *g* for 10 min at 4 °C. The RNA pellet was washed with 1 mL 75% ethanol, vacuum-dried, and dissolved in 50 μL of RNase-free H_2_O. RNA integrity was evaluated using an Agilent 2100 Bioanalyzer (Agilent Technologies, Santa Clara, CA, USA), and RNA concentration was determined using a NanoDrop 2000 spectrophotometer (Thermo Scientific, Waltham, MA, USA). The cDNA libraries were constructed using the VAHTS Universal V6 RNA-seq Library Prep Kit (Vazyme, Nanjing, China) according to the manufacturer’s instruction and sequenced on the llumina Novaseq 6000 platform (150 bp paired-end).

Raw reads were processed using the Fastp program to remove adapter sequences, N-containing and low-quality reads [49]. The clean reads were mapped to the *N. sylvestris* reference genome [24] using HISAT2 software (2.2.0) with default parameters [50]. Transcript abundance was quantified using HTSeq 2.0 [51], and expression levels were normalized to fragments per kilobase of transcript per million fragments mapped reads (FPKM). Principal component analysis (PCA) was performed on the FPKM values of all samples using the PRCOMP function in R. Differentially expressed genes (DEGs) were identified using DESeq2 with the criteria of |log2(FoldChange)| > 1 and q-value < 0.05 [52]. Kyoto Encyclopedia of Genes and Genomes (KEGG) pathway enrichment analysis was conducted using KOBAS 2.0 [53].

### 4.7. Untargeted Metabolome Analysis Through Ultra Performance Liquid Chromatography–Mass Spectrometry (UPLC-MS)

Leaf and flower samples were collected from five-month-old plants, frozen immediately in liquid nitrogen, and ground into a fine powder. For metabolite extraction, the sample (100 mg) was transferred to a 2 mL tube and extracted with 0.8 mL of 75% (*v*/*v*) methanol for 1 h at 4 °C. Following centrifugation at 13,000× *g* for 10 min at 4 °C, the supernatant was filtered through a 0.22-μm membrane. The quality control (QC) sample was prepared by mixing equal aliquot from all samples.

Untargeted metabolomic profiling was performed by using an Acquity UPLC I-Class Plus System (Waters Corp., Milford, MA, USA) coupled to a Q-Exactive Focus mass spectrometer (Thermo Fisher Scientific, Waltham, MA, USA). The chromatographic separation was performed on a HSS T3 C18 reversed phase column (2.1 mm × 100 mm, 1.8-μm particle size; Waters) maintained at 40 °C. The mobile phases consisted of 0.1% formic acid in water (Solvent A) and 0.1% formic acid in acetonitrile (Solvent B). The gradient elution program was as follows: 0.5% B hold for 1 min, linearly increase to 30% B over 15 min, increase to 80% B over 5 min, and return to initial conditions over 3 min. The flow rate was 400 μL min^−1^, and the injection volume was 3 μL. The sheath gas flow rate was 35 arbitrary units (Arb), the auxiliary gas flow rate was 8 Arb, the capillary temperature was 320 °C, and the spray voltage was +3800 V (positive ion mode) or −3000 V (negative ion mode). Mass spectra were acquired in full-scan mode over the mass range of *m*/*z* 100–1200 at a resolution of 60,000, with 3 scans per second. Data were collected in both positive and negative ionization modes. Raw data acquisition was performed using XCALIBUR software (Thermo Fisher Scientific, Waltham, MA, USA).

Mass spectral features were extracted using Progenesis QI v2.4 software (Nonlinear Dynamics Ltd., Newcastle, UK). Peak alignment was performed using the pooled QC sample as reference, normalized to internal standard. The resultant peak matrix from positive and negative modes were exported and merged. The relative peak intensities were calculated for each sample and used for downstream statistical and annotation analyses. The principal component analysis (PCA) was performed using SIMCA 14 software (Umetrics, Umeå, Sweden). The partial least squares discriminant analysis (PLS-DA) was performed to calculate the variable importance in projection (VIP) values. Metabolite annotation was performed by matching accurate masses and fragment ions against compound databases, including Human Metabolome Database (HMDB) [54], Lipid Maps [55], Metabolite and Tandem MS Database (METLIN) [56] and Plant Metabolic Network (https://plantcyc.org). Annotated compounds were classified according to HMDB classification criteria.

### 4.8. Terpenoid Extraction and GC-MS Analysis

Leaf samples were collected from five-month-old diploid and tetraploid plants, immediately frozen in liquid nitrogen, and ground into a fine powder. For terpenoid extraction, 200 mg of sample was transferred to a 2 mL glass vial, adding 1 mL of hexane, and vortexed vigorously for 1 min followed by sonication in an ultrasonic water bath for 30 min at room temperature. The samples were centrifugated at 13,000× *g* for 10 min at 4 °C, and the supernatant was transferred into an amber glass vial for GC-MS analysis.

The GC-MS analysis was performed using an Agilent 5977A mass detector coupled to Agilent 7890B gas chromatograph (Agilent Technologies, Santa Clara, CA, USA) equipped with an HP-5MS capillary column (30 m × 0.25 mm, i.d. 0.25 μm; Agilent, Santa Clara, CA, USA). Helium was used as the carrier gas at a constant flow rate of 1.0 mL min^−1^. The injection port was maintained at 250 °C, and 1 μL of sample was injected in splitless mode. The oven temperature program was set as follows: initial 60 °C held for 2 min, ramped to 250 °C at 10 °C min^−1^, then held of 5 min. The mass spectrometer was operated in electron ionization (EI) mode at 70 eV, with the ion source and quadrupole temperatures set to 250 °C. Mass spectra were acquired in scan mode over the mass range of *m*/*z* 50–550. Data acquisition and processing were performed using MassHunter Workstation software (B.08.00, Agilent, Santa Clara, CA, USA).

Metabolite identification was performed by comparing retention time and mass spectra with authentic standards when available (including CBT-diol and cis-abienol). For compounds lacking authentic standards, tentative identification was achieved by matching mass spectra against the NIST 17 mass spectral library (National Institute of Standards and Technology, Gaithersburg, MD, USA), accepting only matches with a similarity score ≥ 80%. Relative quantification of individual terpenoids was performed by integrating peak areas of characteristic fragment ions from extracted ion chromatograms. The total terpenoid content for each sample was calculated as the sum of all integrated peak areas normalized to the fresh weight of the extracted tissue. All analyses were performed on three biological replicates, each with two technical replicates.

## 5. Conclusions

In this study, we demonstrate that autopolyploidization leads to a profound metabolic reprogramming in *N. sylvestris*, characterized by a shift in carbon flux from sucrose biosynthesis to the specialized metabolite class of diterpenoids. Integrated transcriptomic and metabolomic analyses revealed that this shift is underpinned by the coordinated upregulation of the MEP pathway and diterpene synthase genes, alongside the downregulation of sucrose biosynthetic gene *SPS*. The discovery of this ploidy-driven metabolic switch from primary metabolism to specialized metabolism provides a concrete mechanistic framework for understanding polyploidy-driven metabolic innovation. These findings offer a valuable resource and a theoretical foundation for deploying polyploidy as a tool for metabolic engineering and crop improvement, particularly for enhancing the production of high-value natural bioactive metabolites.

## Data Availability

All data are available in the main text. The data that support the findings of this study are openly available in the Genome Sequence Archive (GSA) at https://ngdc.cncb.ac.cn/gsa/, project number PRJCA065881, accessed on 3 June 2028.

## Supplementary Materials

The following supporting information can be downloaded at: https://www.mdpi.com/article/10.3390/plants15142158/s1, Figure S1: Partial least squares discriminant analysis (PLS-DA) of metabolomic profiles of diploid and tetraploid plants. (A) Leaves. (B) Flowers. CK: diploid plants. D7, D10 and D12: autotetraploid lines; Figure S2: Volcano plots of differentially accumulated metabolites (DAMs) between diploid and tetraploid plants. (A–C) Leaves. (D–F) Flowers. CK: diploid plants. D7, D10 and D12: tetraploid plants; Figure S3: Volcano plots of differentially expressed genes (DEGs) between diploid and tetraploid plants. (A–C) Leaves. (D–F) Flowers. CK: diploid plants. D7, D10 and D12: tetraploid plants.

## Abbreviations

The following abbreviations are used in this manuscript:

WGD	whole-genome duplication
TE	transposable element
DEG	differentially expressed gene
TAD	topologically associating domain
MS	Murashige & Skoog
PCA	principal component analysis
PLS-DA	partial least squares discriminant analysis
DAM	differentially accumulated metabolite
KEGG	Kyoto Encyclopedia of Genes and Genomes
GC-MS	gas chromatography–mass spectrum
MEP	methylerythritol phosphate
IPP	isopentenyl pyrophosphate
DMAPP	dimethylallyl pyrophosphate
GGPPS	geranylgeranyl diphosphate synthase
GGPP	geranylgeranyl diphosphate synthase
DXS	1-deoxy-D-xylulose-5-phosphate synthase
MVA	mevalonate
AACT	Acetyl-CoA acetyltransferase
HMGS	3-hydroxy-3-methylglutaryl-Coenzyme A synthase
HMGR	3-hydroxy-3-methylglutaryl-coenzyme A reductase
EAS	5-epi-aristolochene synthase
SPS	sucrose phosphate synthase
GAP	glyceraldehyde-3-phosphate
MOPS	3-(N-morpholino) propanesulfonic acid
PVP	polyvinylpyrrolidone
FPKM	fragments per kilobase of transcript per million fragments mapped reads
UPLC-MS	liquid chromatography–mass spectrometry
QC	quality control
EI	electron ionization
