# Supplementary material for: Polyploidization Redirects Carbon Flux to Diterpenoid Biosynthesis in Nicotiana sylvestris
Source: Plants (Basel). 2026 Jul 13;15(14):2158. doi: 10.3390/plants15142158 (PMC13414626; doi:10.3390/plants15142158)
Supplement: Supplementary file 1 [file plants-15-02158-s001.zip › plants-4386779-supplementary.pdf]

Polyploidization Redirects Carbon Flux to Diterpenoid Biosynthesis in *Nicotiana sylvestris*

Xiuming Wu[+], Kexin Chen[+], Changqing Yang, Yangyang Sun[*], Min Ren[*]

Tobacco Research Institute, Chinese Academy of Agricultural Sciences, Qingdao, China

[*]Correspondence: renmin@caas.cn (M.R.); sunyangyang@caas.cn (Y.S.)

[+]These authors contributed equally to this work

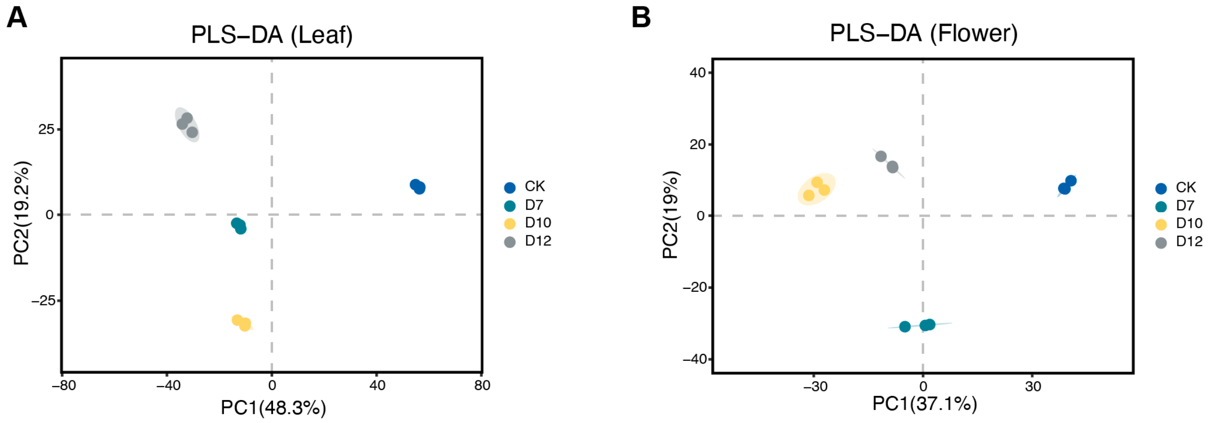

Figure S1. Partial least squares discriminant analysis (PLS-DA) of metabolomic profiles of diploid and tetraploid plants.

(A) Leaves. (B) Flowers. CK: diploid plants. D7, D10 and D12: autotetraploid lines.

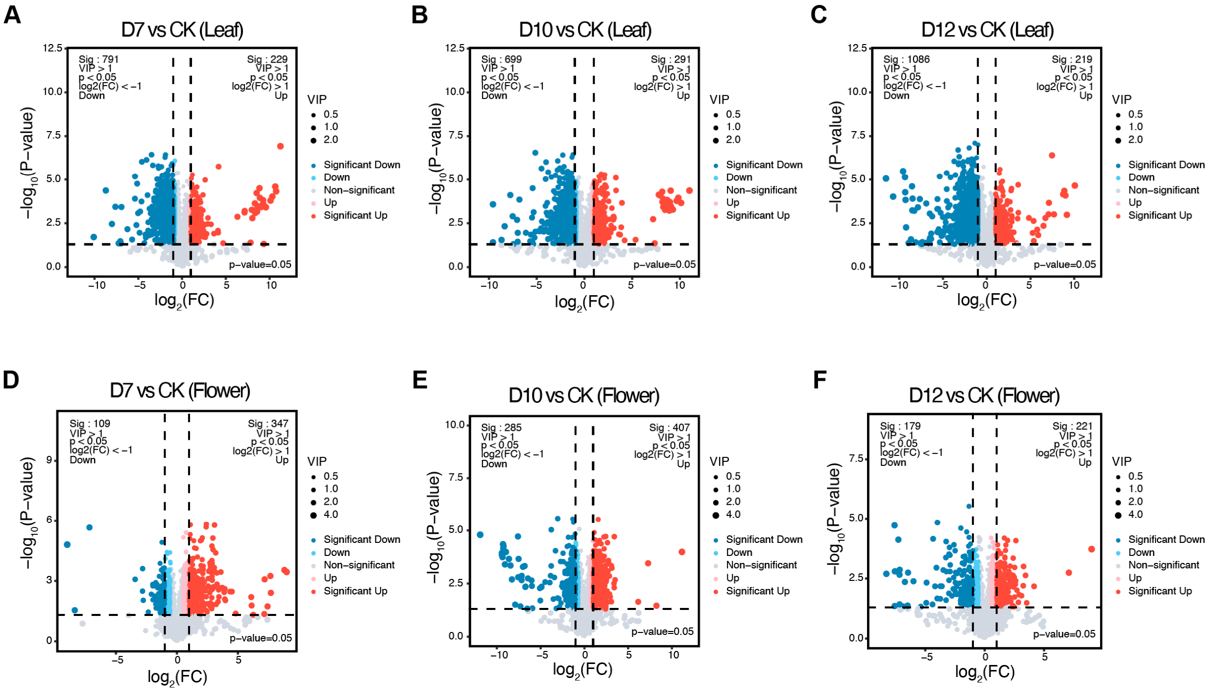

Figure S2. Volcano plots of differentially accumulated metabolites (DAMs) between diploid and tetraploid plants.

(A-C) Leaves. (D-F) Flowers.

CK: diploid plants. D7, D10 and D12: tetraploid plants.

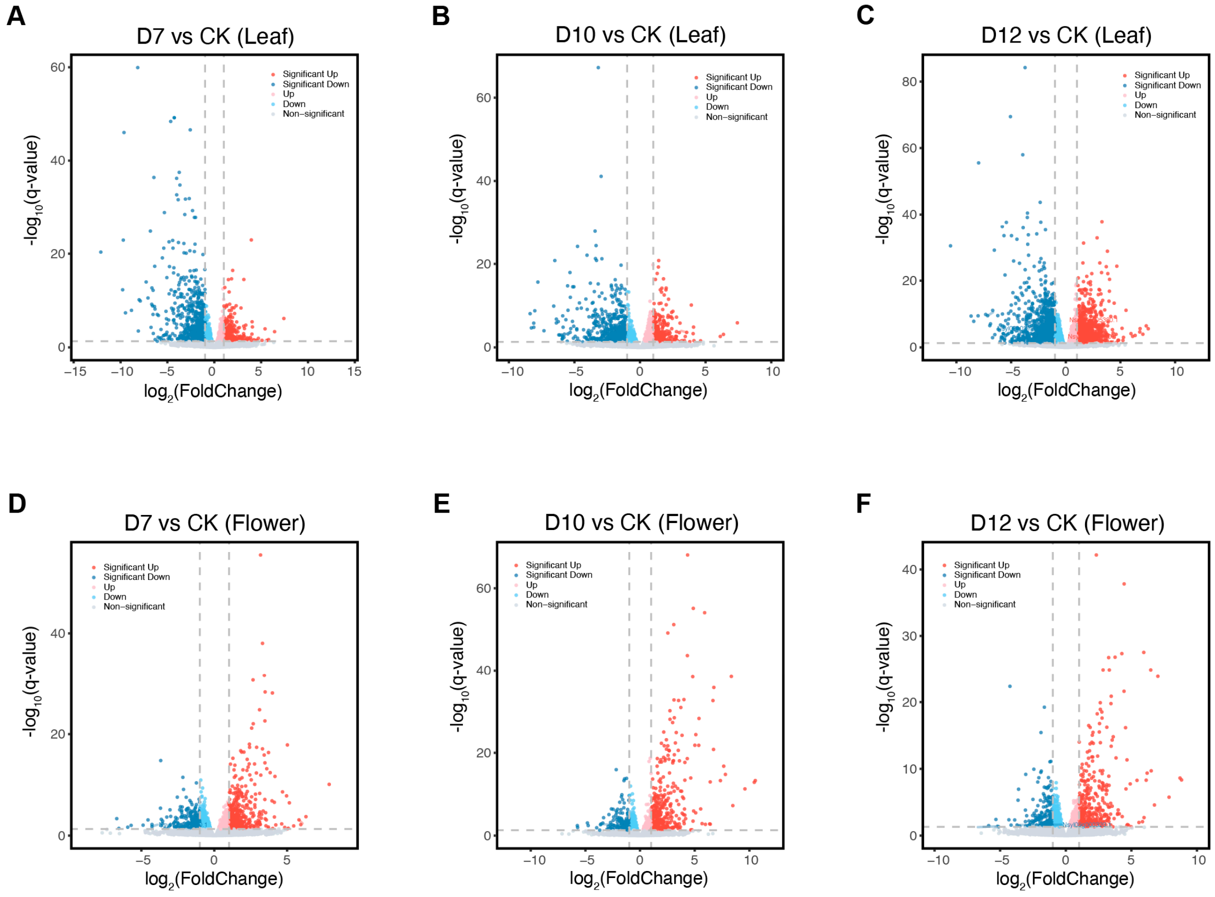

Figure S3. Volcano plots of differentially expressed genes (DEGs) between diploid and tetraploid plants.

(A-C) Leaves. (D-F) Flowers.

CK: diploid plants. D7, D10 and D12: tetraploid plants.
